# Angioedema due to acquired C1 inhibitor deficiency: patient experience, conceptual disease model, and assessment of patient-reported outcome measures

**DOI:** 10.3389/fimmu.2026.1810851

**Published:** 2026-05-14

**Authors:** Mats de Lange, Danny M. Cohn, Delphine Gobert, Marc A. Riedl, Andrea Zanichelli, Kelsie Brewer, Sarah Clifford, Swaha Pattanaik, Beverly Romero, Maggie Chen, Joan Mendivil

**Affiliations:** 1 Department of Vascular Medicine, Amsterdam Cardiovascular Sciences, Amsterdam UMC, University of Amsterdam, Amsterdam, Netherlands; 2 Sorbonne Université, Médecine Interne, AP-HP, Centre de référence des angioedèmes à kinines, Hôpital Saint-Antoine, Paris, France; 3 Division of Allergy and Immunology, University of California, San Diego, La Jolla, CA, United States; 4 Dipartimento di Scienze Biomediche per la Salute, Universita degli Studi di Milano, Milan, Italy; 5 Operative Unit of Medicine, Angioedema Center, IRCCS Policlinico San Donato, Milan, Italy; 6 Sprout Health Solutions, Los Angeles, CA, United States; 7 Pharvaris Inc., Lexington, MA, United States; 8 Pharvaris GmbH, Zug, Switzerland

**Keywords:** angioedema, bradykinin, C1 inhibitor deficiency, patient-reported outcomes, PGI-C

## Abstract

**Background:**

Angioedema due to acquired C1 inhibitor deficiency (AAE-C1INH) is a rare, serious condition that manifests with recurring and often painful swelling attacks. No approved treatments or validated patient-reported outcome (PRO) measures exist for AAE-C1INH. This study aimed to develop a conceptual model of AAE-C1INH and explored the relevance of adapting PRO measures validated for hereditary angioedema, another bradykinin-mediated disease.

**Methods:**

This cross-sectional, qualitative study involved semi-structured interviews with 8 adults living with AAE-C1INH. Open-ended questions elicited participants’ descriptions of AAE-C1INH manifestations and daily life impacts. Cognitive interviews assessed perceptions of clarity, comprehension, and meaningful levels of change in PRO measures: Patient Global Impression of Change (PGI-C) and Severity (PGI-S) and Patient Global Assessment of Status (PGA-S) and Change (PGA-C).

**Results:**

Participants reported traumatic medical emergencies, misdiagnoses, and evaluations by a variety of healthcare providers. Daily life impact was common (social/family and treatment-related; n=7). Attack areas most frequently reported were abdomen (n=7), face (n=6), and foot/hand (n=5 each). Evaluating PGI-C, 8/8 participants correctly interpreted the scale to assess symptoms at a given time post-treatment vs. at time of treatment for a theoretical AAE-C1INH attack. Four of 8 participants considered “a little better” ≥2 hours post-treatment and 6/6 considered “better” ≥4 hours as meaningful. Most also considered PGI-S and PGA easy to interpret and relevant to their symptoms.

**Conclusion:**

Interviews highlighted the impact of AAE-C1INH on participants’ lives and overall well-being. Participants’ assessments of PRO items have informed the clinical outcome assessment strategy for the first phase 3 clinical trial exclusively in AAE-C1INH.

## Introduction

Angioedema due to acquired C1 inhibitor deficiency (AAE-C1INH) has an estimated prevalence of ~1 in 500,000 people, which is estimated to be 10 times rarer than hereditary angioedema (HAE), and is frequently misdiagnosed due to its rarity (1–5). AAE-C1INH is characterized by recurrent attacks of angioedema in different parts of the body, which can become life-threatening if edema affects the upper airways and leads to breathing difficulties (2, 5, 6). AAE-C1INH attacks are often painful, cause discomfort, and limit movement, all of which disrupt daily life and create considerable disease burden (2, 7, 8). Overall, patients with AAE-C1INH have significant unmet medical needs, as no therapies have been approved for either the prevention or treatment of AAE-C1INH attacks (9).

Localized AAE-C1INH swelling attacks result from excessive production of bradykinin that activates bradykinin B2 receptors and causes increased vascular permeability (2). Clinical presentation of AAE-C1INH is similar to that of HAE with C1INH deficiency (HAE-C1INH), although the underlying causes of these bradykinin-mediated diseases differ (9). The main causes of AAE-C1INH are hematological disorders or autoimmune diseases that lead to low C1 inhibitor (C1INH) due to consumption or the presence of anti-C1INH antibodies, whereas most common forms of HAE are associated with C1INH deficiency (type 1) or dysfunction (type 2) resulting from mutations in the *SERPING1* gene (6, 9). In contrast to HAE-C1INH, AAE-C1INH has a negative family history, is generally diagnosed later in life (5, 10), and is often associated with reduced C1q levels (5, 9, 11).

In the absence of treatments indicated for AAE-C1INH, off-label treatment with medications approved for HAE have often been used with variable success (9, 10, 12–16). The bradykinin B2 receptor antagonist deucrictibant is currently being investigated in two phase 3 trials for long-term prophylactic (NCT06669754) and on-demand (NCT06343779) treatment of angioedema attacks in people with HAE (17, 18). In a phase 2 trial, deucrictibant significantly reduced the severity and duration of HAE attacks compared with placebo, and no severe or serious treatment-related adverse events were reported (17). Findings from subsequent investigator-initiated trials in AAE-C1INH were supportive of the potential of deucrictibant to effectively and safely prevent and treat AAE-C1INH attacks (9, 19). Deucrictibant received orphan drug designation from the United States (US) Food and Drug Administration (FDA) in 2022 for the treatment of bradykinin-mediated angioedema (20). More recently, the European Medicines Agency (EMA) granted orphan drug designation to deucrictibant for AAE-C1INH, and the pivotal phase 3 trial CREAATE for prophylaxis and on-demand treatment of AAE-C1INH attacks is planned (NCT07266805).

To evaluate the efficacy of potential treatments for AAE-C1INH attacks in clinical trials, including deucrictibant, there is a need to identify appropriate outcome measures that are relevant to people with AAE-C1INH and endpoints that accurately measure changes in symptoms post treatment. Prior to this study, no patient-reported outcomes (PROs) had been validated in people with AAE-C1INH. Given the clinical similarities of AAE-C1INH with HAE-C1INH, we hypothesized that PRO measurements validated to assess the symptoms and impacts of recurrent angioedema in individuals with HAE (21–24) may also be relevant and suitable in the AAE-C1INH population. To test this hypothesis, we interviewed people with AAE-C1INH to evaluate their comprehension and interpretation of Patient Global Impression of Change (PGI-C) and Severity (PGI-S) and the Patient Global Assessment of Status (PGA-S) and Change (PGA-C), along with other selected clinical outcome assessments. Furthermore, we explored participants’ perceptions of meaningful changes on the PGI-C, PGI-S, PGA-S, and PGA-C measures to support clinically relevant outcomes. To contribute to the considerable knowledge gaps in this disease state, we also aimed to understand participants’ experiences with AAE-C1INH symptoms and the impact on their daily lives in order to develop the first conceptual model of AAE-C1INH.

## Materials and methods

### Study design and participants

A cross-sectional, qualitative interview study included adults with AAE-C1INH living in the US who were asked to answer questions about a theoretical treatment for hypothetical AAE attacks. The study aimed to recruit up to 10 participants who were ≥18 years of age with a diagnosis of AAE-C1INH, ≥1 AAE attack in the last 3 months, and a stable underlying disease (i.e., treatment for the underlying disease causing AAE-C1INH had not changed for the last 3 months). Participants were excluded if they had any prior or concomitant diagnosis of angioedema other than AAE-C1INH. The full list of eligibility criteria is provided in the **Supplementary Material**. The original criteria were amended partway through recruitment to include participants who had experienced ≥1 attack in the last 6 months and those with an unstable underlying disease, owing to the challenges faced in recruiting participants with such a rare condition (**Supplementary Material**).

Recruiting, screening, consenting, and scheduling of participants was carried out by the Hereditary Angioedema Association (HAEA), a US-based patient advocacy group. Study participants were screened using the institutional review board (IRB)-approved screening questionnaire. Upon successful screening, participants completed an informed consent document and provided additional verbal consent to audio-record the interview. The HAEA team pseudonymized participants’ diagnosis forms, screening data, and consent forms prior to sharing them with the study team. Confidentiality of participant records, including encrypted and password-protected files with consented participants’ ID numbers, was protected in accordance with all applicable laws, regulations, and guidelines in the US. Participants’ contact information was only known to HAEA and was not shared with interviewers. Participants received $50 USD to assist with potential costs associated with providing confirmation of diagnosis and $150 USD for contributing to the study upon completion of the interview. The study was approved by the Salus IRB in Austin, Texas, US.

### Interview procedures

Interviewers were trained on the study-specific interview procedures. Interviews were conducted in English, held remotely using video-conferencing software, and lasted approximately 90 minutes, which could be split over one or two sessions depending on participant preference. Interviews used both concept elicitation (CE) and cognitive debriefing (CD) methodology and were undertaken using the IRB-approved semi-structured, combined CE/CD interview guide. Interviewers began by using open-ended CE questions to elicit participants’ diagnoses, daily life experiences, and impacts of AAE-C1INH symptoms. For the remainder of the interview CD methodology was used, and participants were asked to review and discuss a selection of PRO scales to assess their interpretation, clarity, and relevance for use in the AAE-C1INH population (**Supplementary Figure 1**). Participants were also queried about their perceptions of meaningful change on these PRO scales.

The audio recordings of interviews were uploaded to a professional transcription service’s secure, cloud-based platform, which was encrypted and only accessible by pre-approved employees using individually assigned passwords. Audio recordings were transcribed verbatim, sent redacted to the study team for analysis, and then deleted within 14 days of being uploaded.

### Cognitive debriefing of PRO assessments

The PGI-C scale was used to assess the amount of change in AAE-C1INH attack symptoms experienced by participants from the time they first took the medication for a hypothetical AAE attack until “right now,” using a seven-point response scale ranging from “much better” to “much worse” (**Table 1**; **Supplementary Figure 1**). The PGI-S asked participants to assess the current severity of their AAE-C1INH attack symptoms with a five-point response scale ranging from “no symptoms” to “very severe” (**Table 1**; **Supplementary Figure 1**). The PGA-S asked participants to assess the current impact of AAE-C1INH on their overall quality of life (QoL) based on a five-point response scale ranging from “no impact” to “very severe impact” (**Table 1**; **Supplementary Figure 1**). For the PGA-C, participants assessed overall change in health-related QoL (HRQoL) since starting the theoretical medication, using a five-point response scale ranging from “much better” to “much worse” (**Table 1**; **Supplementary Figure 1**).

**Table 1:** Clinical outcome assessments.^a^

Assessment	Description of PRO assessments^b^	Meaningful change interpretation
PGI-C	Self-reported measure that assesses change from baseline in AAE-C1INH attack symptoms since taking medication for the attack. It uses a seven-point verbal rating scale that includes the response options of “much better,” “better,” “a little better,” “same,” “a little worse,” “worse,” and “much worse.”	Participants were asked to discuss, hypothetically, the levels of change they would perceive as meaningful at various time points post treatment of an attack.
PGI-S	Self-reported measure that asks participants to report the current severity of their AAE-C1INH attack symptoms using a five-point verbal rating scale that includes the response options of “no symptoms,” “mild,” “moderate,” “severe,” and “very severe.”	Participants were asked to complete a baseline “pre-treatment” version of the PGI-S, and then to discuss, hypothetically, what levels of change they would perceive as meaningful at various time points post treatment of an attack.
PGA-S- Quality of Life	Self-reported measure that asks patients to assess the current impact of their AAE-C1INH on their quality of life using a five-point verbal rating scale that includes the following response options: “no impact,” “mild impact,” “moderate impact,” “severe impact,” and “very severe impact.”	Participants were instructed to imagine they were participating in a hypothetical 12-week clinical trial for a prophylactic treatment for AAE attacks and to provide a baseline response (representing their status at the start of the trial) and how much change from baseline was required on the assessment tool at the end of the trial to be considered meaningful.
PGA-C- Quality of Life	Self-reported measure that asks patients to assess overall change in how their quality of life has been impacted by AAE-C1INH since first taking the study drug in the hypothetical 12-week clinical trial. It uses a five-point verbal rating scale that includes the response options of “much better,” “a little better,” “no change,” “a little worse,” and “much worse.”	Participants were instructed to imagine they were participating in a hypothetical 12-week clinical trial for a prophylactic treatment for AAE attacks and about the level of change in quality of life they would perceive as meaningful at the end of the 12-week trial.

AAE-C1INH, angioedema due to acquired C1-inhibitor deficiency; PGA-C, Patient Global Assessment of Change; PGA-S, Patient Global Assessment of Status; PGI-C, Patient Global Impression of Change; PGI-S, Patient Global Impression of Severity; PRO, patient-reported outcome.

^a^ Each assessment uses a single-item scale to measure disease severity, impact, or change.

^b^ Participants were asked to provide assessments of a hypothetical AAE attack for each PRO assessment.

### Meaningful levels of change on PRO scales

Participants were asked about potential meaningful levels of change on the PGI-C response scale at 15 minutes, 30 minutes, 60 minutes, 2 hours, 3 hours, 4 hours, 12 hours, 24 hours, and 48 hours post treatment for a hypothetical AAE attack (**Table 1**). For 30 minutes post treatment and beyond, participants were asked to provide only an open-ended response regarding their desired level of change and their view on whether “a little better” and “better” would be meaningful changes. Participants were also asked about potential meaningful levels of change from baseline (pre-treatment score) on the PGI-S response scale to 15 minutes, 30 minutes, 60 minutes, 2 hours, 3 hours, 4 hours, 12 hours, 24 hours, and 48 hours following treatment for an AAE attack (**Table 1**).

For the PGA-S, participants were asked to consider a hypothetical 12-week clinical trial and provide a hypothetical pre-trial baseline response. They were then asked what level of change in impact on QoL they would like to experience at the end of the trial. (**Table 1**). In a similar fashion for the PGA-C, participants were asked to provide a hypothetical response as if they had participated in a 12-week clinical trial and qualify whether their response was a level of change in QoL they would consider meaningful (**Table 1**).

### Qualitative analysis of concept elicitation data

The Master Coder (MC) from the research team developed the first version of the coding framework in MAXQDA 24, a qualitative analysis software program (VERBI Software, 2024). The MC then trained all study team analysts on this coding framework and related procedures. Inter-coder reliability was evaluated through a process known as “double-coding,” which involved regular discussions between multiple coders to align on their interpretation of data. The coding framework was modified as necessary to ensure consistency of coding.

All CE data from part 1 of the interviews were coded and analyzed using principles of thematic analysis and additional features from grounded theory (25–28). This approach conforms to best practices in the clinical outcome assessment field (29). Analysts read and re-read transcripts to identify themes, attributes, concepts, and relationships emerging from interviews. Once a draft coding framework was developed, reviewed, and coders were selected and trained, the inter-coder reliability process took place. This process (“double-coding”) confirmed that coders were approaching interpretation of the data in a consistent manner. Two trained coders independently coded the content of the first three transcripts using the coding framework. A comparison of coding results from these transcripts helped ensure consistency of coding through identification and resolution of areas of disagreement or uncertainty in the application of codes to the transcript segments. Coding discrepancies were reconciled by modification or re-definition of the coding framework, and any revisions that were made to the coding framework to accommodate new data were reviewed and discussed by both coders, with leadership of the process by the MC.

A saturation grid of concepts related to AAE-C1INH attacks as reported by participants was developed. Saturation is said to be reached when no new or important concepts emerge from the data (30). Finally, the CE data were used to develop a conceptual model of AAE-C1INH.

### Qualitative analysis of cognitive debriefing data

Cognitive interview data were analyzed using a content analysis approach with a focus on item-level analysis and identification of issues associated with interpretation, recall, and clarity. Relevance to the participant’s experience of AAE-C1INH was also assessed. An initial coding framework based on the interview guide was developed in MAXQDA. The coding framework was refined, and new codes were added when needed throughout the coding process.

### Descriptive statistics

Descriptive statistics were used to summarize data and are presented as both frequencies and percentage of the total sample.

## Results

### Demographics

Of 169 people with AAE who were approached to participate, eight met the inclusion criteria and took part in the qualitative interviews (**Table 2**). All participants identified as White, and most (n=7, 87.5%) were female. Education level was generally high among the participants; 75.0% (n=6) had a bachelor’s degree or higher and all reported attending at least “some college.” Over half of participants (n=5, 62.5%) experienced symptom onset between the ages of 50 and 60 years (mean, standard deviation [SD]: 55.9 [7.1] years), and the mean (SD) age at diagnosis was 57.0 (9.4) years. The most frequently reported underlying condition was monoclonal gammopathy of clinical significance (n=3, 37.5%).

**Table 2:** Participant demographics and characteristics.

Characteristics	Participants, N = 8
Mean age (SD) [range], years	67.6 (9.2) [48-77]
Mean age at symptom onset (SD) [range], years	55.9 (7.1) [48-68]
Mean age at diagnosis (SD) [range], years	57.0 (9.4) [48-68]
Mean number of attacks in past 12 weeks (SD) [range]	5.5 (10.0) [0-30]
Sex, n (%)	
Female	7 (87.5)
Male	1 (12.5)
Race/Ethnicity, n (%)	
White	8 (100)
Highest education level achieved, n (%)	
Some college	1 (12.5)
2-year associate degree	1 (12.5)
4-year bachelor’s degree	2 (25.0)
Master’s degree	3 (37.5)
Doctoral or professional degree	1 (12.5)
Treatments prescribed for AAE-C1INH^a^, n (%)^b^	
Icatibant (30 mg/3 mL, as needed)	7 (87.5)
Berotralstat (150 mg, once a day)	2 (25.0)
Lanadelumab (300 mg/2 mL, every 2 weeks)	2 (25.0)
C1 esterase inhibitor-human (unknown dosage, before dental procedures)	1 (12.5)
Danazol (unknown dosage, before dental procedures)	1 (12.5)
C1 esterase inhibitor-recombinant (2100 units, before medical procedures)	1 (12.5)
Underlying conditions, n (%)	
Monoclonal gammopathy of clinical significance	3 (37.5)
Myasthenia gravis	1 (12.5)
Breast cancer (in remission)	1 (12.5)
Chronic lymphocytic leukemia	1 (12.5)
Lymphoma workup negative	1 (12.5)
Lymphoproliferative B-cell disorder	1 (12.5)

AAE-C1INH, angioedema due to acquired C1-inhibitor deficiency; SD, standard deviation.

^a^ Prescribed off-label for AAE attacks by physicians.

^b^ Participants may have been prescribed more than one treatment.

### Concept elicitation findings

#### Diagnosis experience and healthcare resource use

Most participants reported traumatic medical emergencies and multiple misdiagnoses such as cellulitis, alcohol intoxication, bug bites, medication side effects, and conversion disorder before receiving a diagnosis of AAE-C1INH.

“I was diagnosed in 2019. I am a [mentions a specific health care role], and I diagnosed myself. I had four hospitalizations that year and – so, you know, big facilities and no-one knew what I had, and I finally diagnosed myself.” [Participant 4]

The impact of misdiagnosis among participants who were coping with unexplained, unpredictable, and often severe AAE-C1INH symptoms could be profound, as one participant explained:

“I mean, it’s a horrible experience. I really feel like I have medical PTSD [post-traumatic stress disorder], but, like, in some ways, like, being intubated, it’s like at least they take me seriously now, right? Or being intubated twice, at least they take me seriously now.” [Participant 3]

When participants were asked about healthcare resource utilization, six participants (75%) noted that they visited an allergist/immunologist at some point during their journey to diagnosis. Visits to the emergency department were common and discussed by six participants (75%), all of whom had visited the emergency department when they experienced their first AAE-C1INH attack and were uncertain of the diagnosis. Three of these participants reported no further emergency visits after their first AAE-C1NH attack. Half of participants (n=4) reported difficulties with healthcare providers (HCPs) and highlighted an absence of support or inadequate knowledge about AAE-C1INH. Additionally, all eight participants shared that the treatments they received for their attacks were HAE medications not approved for use in individuals with AAE-C1INH.

#### Attack areas

The number of attacks in the 12 weeks prior to the study was variable (overall mean [SD]: 5.5 [10.0]); one participant experienced no attacks yet did report two attacks in the previous 24 weeks. Another participant experienced 30 attacks in the previous 12 weeks. The other six participants reported between one and five attacks in the 12 weeks prior to study enrollment.

**Figure 1:**
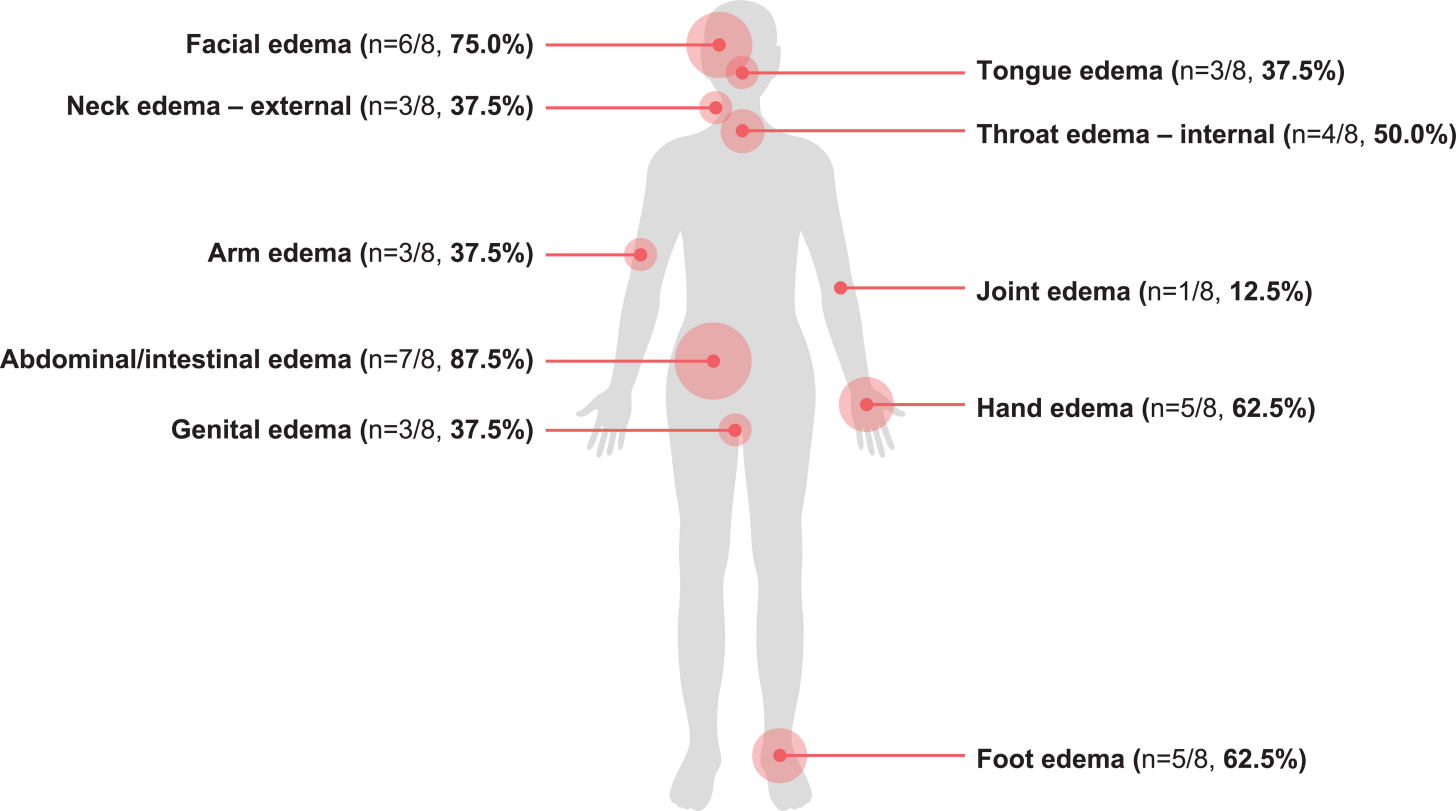
Unique attack areas described by participants during AAE-C1INH attacks. AAE-C1INH, angioedema due to acquired C1-inhibitor deficiency.

Participants identified 10 unique attack areas, with abdominal/intestinal edema (n=7, 87.5%) and facial edema (n=6, 75.0%) reported most frequently (**Figure 1**). Detailed descriptions of each attack area are provided in the **Supplementary Material**. For each attack event, participants described various event-specific physical symptoms and related physical impacts, referred to as subconcepts (**Supplementary Table 1**). Overall, 57 different subconcepts were reported across all participants and attack areas, with a mean of 11.6 subconcepts reported per participant. All 10 attack areas were reported by the fifth interview; however, new subconcepts were still reported in the last two interviews, indicating that saturation on subconcepts was not reached and further interviews may be warranted. By the fifth interview 80.7% of subconcepts were reported, and by the seventh interview 94.7% were reported.

Five of the eight participants provided rankings of the reported symptoms/attacks they would most like to improve. All five ranked abdominal/intestinal edema in the top three, and foot, hand, and facial edema were selected by one participant each.

#### Impacts on daily life

**Figure 2:**
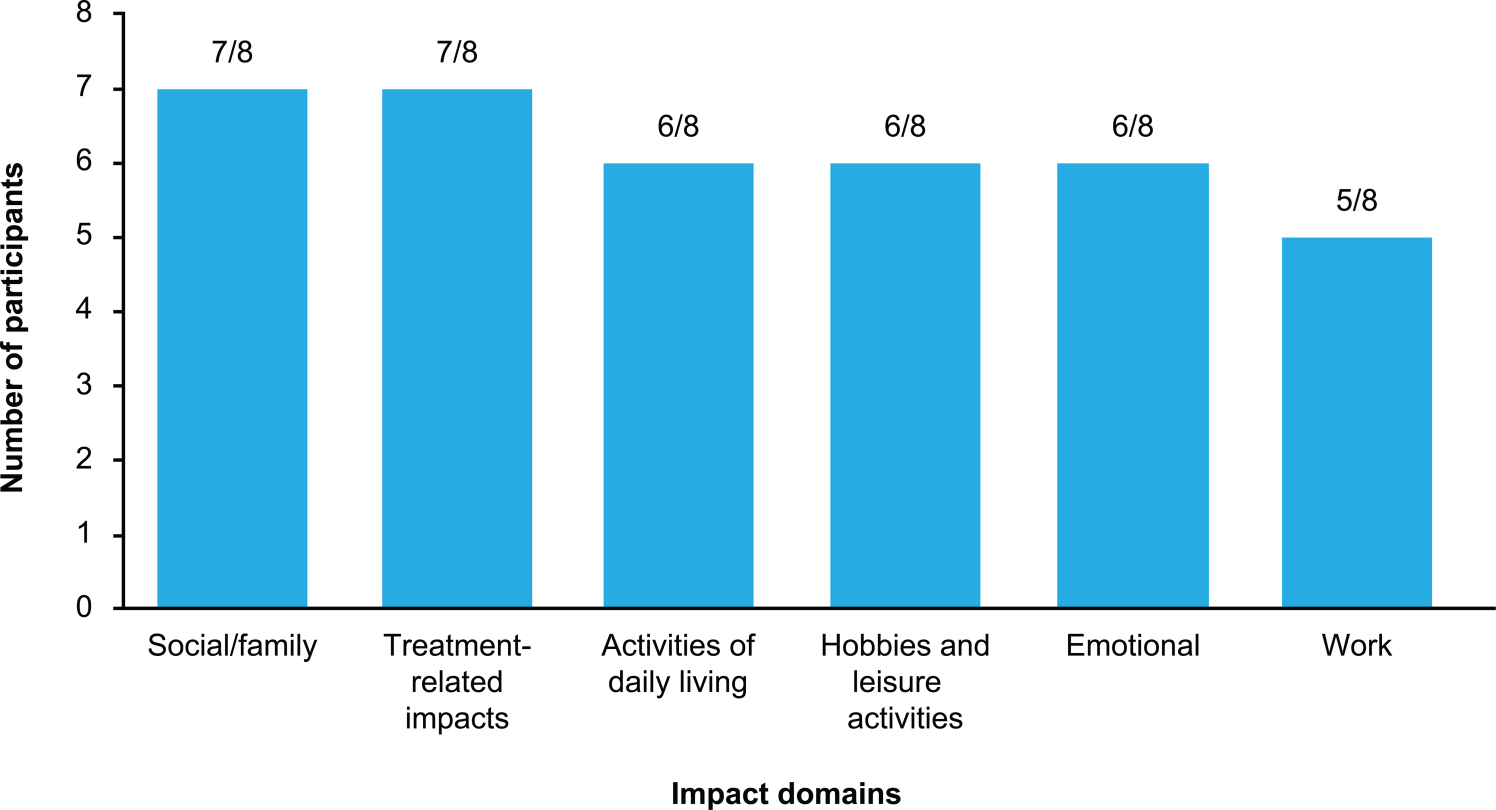
Reported impacts from living with AAE-C1INH across impact domains. AAE-C1INH, angioedema due to acquired C1-inhibitor deficiency.

Participants reported a variety of ways in which AAE-C1INH impacted on their daily lives, which spanned six different domains (**Figure 2**).

#### Social and family impacts

Most participants (n=7, 87.5%) reported at least one social- or family-related impact. The most frequently reported impact (n=5, 62.5%) was “Don’t want to go out/leave home,” with a participant citing their appearance as a key factor:

“Because in life, you go out, you see people, you go to work, you do this, you do that and all of that is delayed or hampered or – I mean, maybe there are people that could go, I’m not one of them. I – there’s just no way. I mean, you look like a freak and (sic) like a true freak.” [Participant 7]

Other themes in this domain reported by one or two participants were: Less support available for AAE than HAE, Family and friends worry, Missed big life events/holidays, Spouse must care for them, Family didn’t grasp severity, Lives with parents as feels unsafe to live alone, and Needs help caring for child.

#### Treatment-related impacts

Treatment-related impacts were reported by nearly all participants (n=7, 87.5%). The most frequently reported were related to the high cost of medication and challenges with insurance coverage for treatments (each noted by three participants [37.5%]):

“The [mentions a specific medication] shots, even though they’re generic, are very expensive and I – obviously, I don’t take one if just my foot or my hand is swelling, but the facial ones you have to be concerned.” [Participant 1]

“And so, it usually involves getting a prescription from the allergist, then the regular insurance has to turn it down, not once, but twice, and then, you know, I’m eligible for two different patient assistance programs….” [Participant 5]

Other treatment-related impacts reported by two or more participants included: Need to use medications ahead of procedures/high-risk events, Difficulty explaining AAE or medications to other HCPs (both reported by three participants [37.5%]), HCP’s lack of support/knowledge to get medications, and Needing to treat with multiple shots (both reported by two participants [25%]).

#### Activities of daily living

Six participants (75.0%) reported that living with AAE-C1INH impacted their activities of daily living. The most frequently reported impact was “Can’t do anything/have to cancel plans” when they are experiencing an attack (n=3, 37.5%):

“Well, if I have an abdominal attack, I’m in bed, completely in bed. My husband has to take care of me. I don’t go to work the next day, or I don’t do any – I have to cancel everything I had planned. Even though I’m not having pain, I just am so wiped out.” [Participant 6]

Other impacts reported included being “Unable to drive,” (n=2, 25.0%) and that they “Can’t write” (n=2, 25.0%). The impacts were broad, and participants reported that attacks “Impact clothing choices” (n=1, 12.5%) and make it “Difficult to put laundry away” (n=1, 12.5%).

#### Hobbies and leisure activities

Six participants (75%) reported impacts of AAE-C1INH on their hobbies and leisure activities, most notably on their ability to travel (n=5, 62.5%) owing to fears of experiencing an attack while away from home and the associated logistics of accessing medication or canceling plans:

“So, I retired early, you know, for instance, and we’re careful about travel. So, before I got on the oral medication, we were really careful about where we traveled, so it, kind of, keeps us from doing that, and I think it affects your activities in a lot of ways. So, you don’t have the same vitality that you – that I did before this disease.” [Participant 4]

“I had to cancel plans for travel. I’m a person who is a camper. I had to cancel camping trips because I didn’t know if I had to find a hospital. It’s really severe.” [Participant 6]

Other participants described limitations on activities such as fishing, dancing at weddings, gardening, and volunteering in their community.

#### Emotional impacts

All but two participants (n=6, 75.0%) reported experiencing ≥1 emotional impact related to living with AAE-C1INH. The most frequently reported impacts were “Stress related to treatment” and “Fear of having a potential attack,” each reported by three participants (37.5%). Treatment-related stress stemmed from trying to determine whether they were having an attack and should administer treatment. The high cost of treatment and difficulty obtaining it exacerbated the concern and decision-making process:

“Because this medicine’s so precious and it’s expensive and you only have so many doses, and I think that just exacerbates it mentally…” [Participant 8]

Two participants reported fear of a potential attack affecting their throat or larynx, with one describing:

“And I was always petrified, too, because the attacks could happen in your larynx and they can happen in your mouth and, you know, if you Google it, you see these pictures of people with, you know, the swollen [larynx] – and, you know, and it’s always ‘Be careful, be careful,’ you know, ‘if it happens in your larynx.’ I was petrified, you know…” [Participant 2]

Other emotional impacts included “Embarrassment” associated with experiencing an attack and “Difficulty dealing with and accepting the diagnosis” (n=2 for both, 25%). There were several other specific mental health and emotional impacts each reported by one participant (12.5%), including Bothered by dependency on medication, Feels “traumatized” by medical experiences, Affects whole “psyche,” Worry when having throat edema, “don’t want to live like this,” Mentally/emotionally “debilitating and devastating,” and Feels vulnerable and older than actual age.

#### Work impacts

Five participants (62.5%) reported a variety of impacts of AAE-C1INH on their employment, with the most frequently reported being “Missed work” (n=3, 37.5%), followed by “Swelling impacts ability to complete job tasks” (n=2, 25%) and two (25.0%) who avoid the workplace and choose to “Work from home instead of office.”

“I have to remove myself and take care of it (the attack) and I’m not going out, I couldn’t work, I couldn’t do anything except treat it.” [Participant 6]

Other impacts reported by one participant (12.5%) each included: “Retired early,” “Had attack at work,” “Difficulty concentrating at work,” “Self-conscious about appearance at work,” and “Avoids going to lunch.”

#### Conceptual model

**Figure 3:**
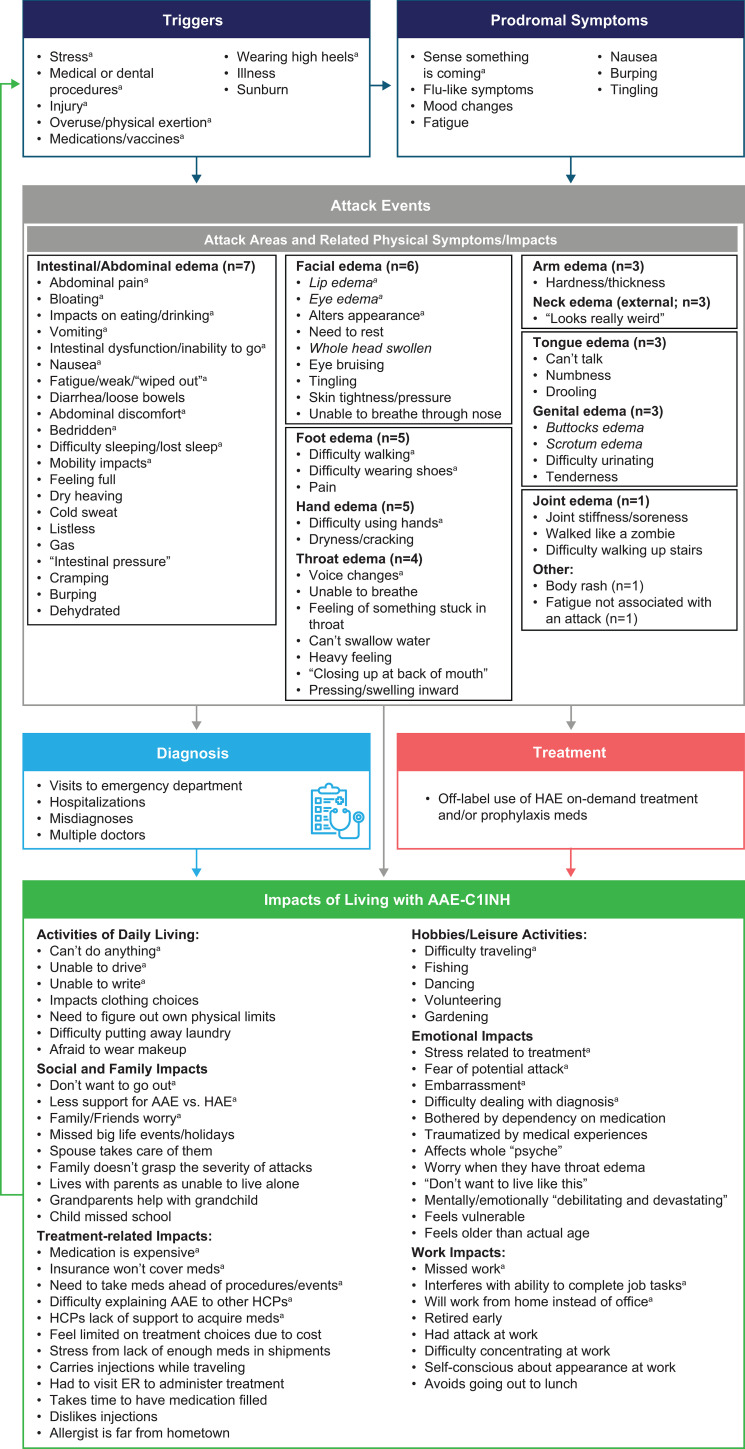
Full conceptual model of AAE-C1INH. ^a^Concepts reported by two or more participants. AAE-C1INH, angioedema due to acquired C1-inhibitor deficiency; ER, emergency room; HAE, hereditary angioedema; HCPs, healthcare professionals; LTP, long-term prophylaxis; ODT, on-demand treatment.

Participant insights and analyzed interview data were used to develop a conceptual model of AAE-C1INH (**Figure 3**). Overall, this visual model highlights the relationship between the more proximal manifestations of AAE-C1INH and the more distal impacts on daily life activities and overall QoL.

### Cognitive debriefing findings

#### Evaluation of PRO assessments

In the CD portion of the interview, participants completed and discussed the PGI-C, PGI-S, PGA-S, and PGA-C items with interviewers. Of the eight participants, all completed the PGI-C, seven participants completed PGI-S and PGA-S, and six participants completed PGA-C in the allotted interview time.

#### Ease of interpreting and answering PRO assessments

Overall, most participants correctly interpreted the PGI-C, PGA-S, and PGA-C. However, four participants reported some difficulty interpreting the PGI-S, such as uncertainty about the time period to consider when answering the question, and how to determine which severity levels to select as certain symptoms may have different severity ratings during the course of an attack (**Table 3**). Similarly, most participants found the PGI-C, PGA-S, and the PGA-C easy to answer; however, only about half of participants (n=4, 57.1%) found the PGI-S easy to answer. Reported challenges included needing a longer time period to accurately describe the disease severity and discerning varying severity levels of one or more symptoms.

**Table 3:** Cognitive debriefing: participants’ interpretation and degree of understanding of each PRO measure.

PRO measure	Correctly comprehended and interpreted response scale options	Reported tool was easy to understand and answer
PGI-C	100% (8/8)	75.0% (6/8)
PGI-S	42.9% (3/7)	57.1% (4/7)
PGA-S	86.0% (6/7)	86.0% (6/7)
PGA-C	83.3% (5/6)	71.4% (5/7)

PGA-C, Patient Global Assessment of Change; PGA-S, Patient Global Assessment of Status; PGI-C, Patient Global Impression of Change; PGI-S, Patient Global Impression of Severity; PRO, patient-reported outcome.

#### Meaningful changes on the PGI-C scale

All eight participants completed the PGI-C and reported that feeling “a little better,” “better,” or “much better” measured 15 minutes post treatment would represent a meaningful change in AAE attack symptoms (**Table 4**).

**Table 4A:** Participant perception of the meaningfulness of a change of “a little better” in PGI-C rating at different time points post treatment.

Query: was “a little better” a meaningful change at this time point?									
Participant	15 min	30 min	60 min	2 h	3 h	4 h	12 h	24 h	48 h
1	√	√	√	√	√	√	x	x	x
2	√	√	√	√	√	√	√	√	x
3	√	√	x	x	x	x	N/A	N/A	N/A
4	√	√	√	x	x	x	x	x	x
5	√	√	x	x	N/A	N/A	x	N/A	N/A
6	√	?	x	x	N/A	x	N/A	?	x
7	√	√	√	√	√	√	√	√	√
8	√	√	√	√	x	x	x	x	x
n (%) who found PGI-C “a little better” meaningful	8/8 (100.0)	7/8 (87.5)	5/8 (62.5)	4/8 (50.0)	3/6^a^ (50.0)	3/7^b^ (42.9)	2/6^a^ (33.3)	2/6^a^ (33.3)	1/6^a^ (16.7)

^a^Two participants were not asked.

^b^One participant was not asked.

Please note additional information for certain participants:

Participant 2 said “yes” at 12 h but “less meaningful” at 24 h.

Participant 3 started to think that any swelling would be a new episode for them at 12, 24, and 48 h.

Participant 5 said “yes, if given information that helped me see that it will take more than ‘30 minutes’ before it reaches full effectiveness.”

Participant 6 gave an unclear response at 30 min, stating that “a little better” was “not convincing” and they preferred the term “better”; at 12 h, they were not “not asked” as they said that symptoms resolved with [named pharmaceutical], “so would like to see much better”; at 24 h their response was unclear, as it depends on “factors like costs, if it’s awkward to inject or take, if it has to be taken for rest of their life.”

Participant 7 at 12 h: they said “yes” but “less meaningful.”

N/A, not asked; participants did not provide an answer in these cases; PGI-C, Patient Global Impression of Change; a “√” indicates the participant found the time point meaningful; a “x” indicates the participant did not find the time point meaningful; a “?” indicates the participant was not sure of that time point’s meaningfulness.

The number of participants who reported that “a little better” was meaningful declined over time, particularly at 2 hours and beyond (**Table 4A**). At 30 minutes, seven participants (87.5%) considered it meaningful, and at 60 minutes the number declined to five participants (62.5%). Two participants were not asked about the meaningfulness of “a little better” at 3 hours and beyond, as they had made it clear that a more significant improvement was required from 2 hours onward.

All eight participants found “better” to be meaningful up to 2 hours post treatment, and most found it meaningful up to 4 hours. (**Table 4B**). One participant (Participant 3) was not asked about a few of the later time points, as they believed if symptoms persisted for 12 hours or longer they would consider it a new episode, and any treatments taken the previous day would no longer be effective in managing the continued symptoms. At 3 hours post treatment, five participants (Participants 2, 3, 5, 7, and 8) initially reported that the change would need to be “much better” to be meaningful (**Table 4C**), but upon probing, four of these participants (Participants 2, 5, 7, and 8) agreed that “better” would also be meaningful (**Table 4B**).

**Table 4B:** Participant perception of the meaningfulness of a change of “better” in PGI-C rating at different time points post treatment.

Query: was “better” a meaningful change at this time point?									
Participant	15 min	30 min	60 min	2 h	3 h	4 h	12 h	24 h	48 h
1	√	√	√	√	√	√	x	x	x
2	√	√	√	√	√	√	√	√	x
3	√	√	√	√	x	N/A	N/A	N/A	N/A
4	√	√	√	√	√	√	x	x	x
5	√	√	√	√	√	N/A	x	N/A	x
6	√	√	√	√	√	√	N/A	x	?
7	√	√	√	√	√	√	√	√	√
8	√	√	√	√	√	√	√	x	x
n (%) who found “better” meaningful	8/8 (100.0)	8/8 (100.0)	8/8 (100.0)	8/8 (100.0)	6/7 (87.5)	6/6^a^ (100.0)	3/6^a^ (50.0)	2/6^a^ (33.3)	1/7^b^ (14.3)

^a^Two participants not asked.

^b^One participant not asked.

Please note additional information for certain participants:

Participant 3 – At 12 h, this participant thought of this as a new attack.

Participant 6 – At 48 h, this participant gave an unclear response, noting that “much better” would be meaningful and “better” would be “maybe at some point” but did not specify at what point.

N/A, not asked; participants did not provide an answer in these cases; PGI-C, Patient Global Impression of Change; a “√” indicates the participant found the time point meaningful; a “x” indicates the participant did not find the time point meaningful; a “?” indicates the participant was not sure of the meaningfulness of that time point.

**Table 4C:** Participant perception of the meaningfulness of a change of “much better” in PGI-C rating at different time points post treatment.

Query: was “much better” a meaningful change at this time point?									
Participant	15 min	30 min	60 min	2 h	3 h	4 h	12 h	24 h	48 h
1	√	N/A	N/A	N/A	N/A	√	√	√	√
2	√	√	√	√	√	√	√	√	√
3	√	N/A	√	√	√	√	√	N/A	N/A
4	√	N/A	√	√	N/A	N/A	√	√	√
5	√	√	√	N/A	√	√	√	√	√
6	√	N/A	√	√	N/A	N/A	√	√	√
7	√	√	N/A	√	√	√	√	√	√
8	√	N/A	√	√	√	√	√	√	√
n (%) of participants who found “much better” meaningful	8/8 (100.0)	3/3 (100.0)	6/6 (100.0)	6/6 (100.0)	5/5 (100.0)	6/6 (100.0)	8/8 (100.0)	7/7 (100.0)	7/7 (100.0)

N/A, not asked or participant did not provide a response; PGI-C, Patient Global Impression of Change; a “√” indicates the participant found the time point meaningful.

When asked about later time points post treatment, more participants were uncertain or indicated that “a little better” and sometimes “better” were no longer meaningful changes. Only three of seven participants (42.9%) thought “a little better” was meaningful at 4 hours. At 12 hours, only two of six participants (33.3%) thought “a little better” was meaningful, and half of participants (50%) thought “better” was meaningful.

“…anything that’s moving me toward the right direction of not having a full-blown swelling, whether it’s defined as ‘better’ or ‘much better,’ is just good.” [Participant 8, at 4 hours]

When asked about expectations for 12 hours post treatment, all participants expected to feel “much better” and expected the swelling to have “dissipated” or “reabsorbed.”

#### Meaningful changes on the PGI-S scale

Seven participants provided answers on meaningful levels of change from baseline (pre-treatment) on the PGI-S scale. Two participants (Participants 5 and 8) reported a “mild” severity, four (Participants 1, 2, 3, and 4) reported “moderate” severity, and one (Participant 7) reported “severe” symptoms at baseline. All participants desired some level of change 30 minutes post treatment. Five participants (71.4%) thought one level of change would be meaningful, and 2/7 (28.6%) reported that two levels were required for meaningful change. At 12 hours post treatment, most participants (Participants 1, 3, 4, and 7) desired at least two levels of change to consider the improvement meaningful. At 24 hours post treatment, regardless of their initial baseline response, all seven participants reported that “no symptoms” was the only meaningful level of change.

#### Meaningful changes on the PGA-S scale

Of the seven participants who reviewed the PGA-S to assess the impact of AAE on their overall QoL, two participants (28.6%) reported “moderate impact,” three participants (42.9%) reported “mild impact,” and two participants (28.6%) reported “very severe impact” at baseline. Of the six participants who provided accurate interpretations of the PGA-S response scale and understood the meaningful change questions, three (50.0%) reported that they would find it meaningful if their QoL impact was stable and unchanged after 12 weeks. One participant (Participant 7) wanted one level of change in their AAE-C1INH impact after 12 weeks, and the two participants who answered “very severe impact” at baseline wanted their impact level to improve by two levels at the end of 12 weeks.

At the group level, the overall average level of change in the impact of their disease on their QoL expected after 12 weeks of treatment increased with the level of impact they reported currently experiencing. For example, participants who started at “moderate impact” said they would expect a level of change of 1.0 on average, whereas those starting at “very severe impact” said they would expect a level change of nearly two points (1.8).

#### Meaningful changes on the PGA-C scale

All six participants who discussed the PGA-C reported that a change in QoL to “much better” after 12 weeks in a hypothetical clinical trial would be a meaningful improvement in QoL, and all but one participant (n=5, 83.3%) reported that a change to “a little better” would also be meaningful. One participant (Participant 4) said that “no change” in QoL would be meaningful if the new medication did not require refrigeration, as is the case with their current regimen. All six participants thought that “a little worse” would be meaningful to them in a negative or “a disappointing sense” (Participant 2).

## Discussion

AAE-C1INH is a serious condition that remains poorly understood, and more research is needed to inform clinical trials investigating potential therapies for this rare disease. To address this research gap, this study interviewed eight participants with AAE-C1INH, and their rich descriptions of symptoms, experiences, and impacts informed a new patient-centered conceptual model for AAE-C1INH. During cognitive interviews participants shared that the PGI and PGA items were relevant, easy to interpret, and provided valuable insights into what may constitute a meaningful change on each scale at different post-treatment time points. Altogether, these findings may inform a clinical outcome assessment strategy for clinical trials in individuals with AAE-C1INH.

The study highlighted the diverse and unique unmet needs of individuals living with AAE-C1INH. Participants reported being unable to engage in normal daily activities, a lack of support and understanding, fear of the unpredictability of attacks, difficulty traveling, work disruptions, and expensive treatments that were not always covered by insurance. Participants also discussed frequent misdiagnoses and consulted multiple physicians before they received the correct diagnosis. This is a common challenge identified in retrospective and survey studies that report varying times to reach a diagnosis of AAE-C1NH (4–6, 13, 31), with the shortest reported median time to diagnosis of 7.5 months (5) and the longest a mean of 5 years (4). Such delays mean that people with AAE-C1INH may experience debilitating symptoms for months or years before they receive appropriate treatment (5). Moreover, all participants in this study were receiving treatments indicated for HAE, which were not developed or approved for treatment of AAE-C1INH.

Of all PROs assessed, PGI-C was the only scale that was understood by all participants. A PGI-C rating of “a little better” was deemed a sufficient improvement for all participants at 15 minutes post treatment and most participants at 30 minutes; however, half of the participants considered this response meaningful at 2 hours post treatment. Overall, a PGI-C rating of “better” was most consistently deemed meaningful across all participants at time points of up to 4 hours post treatment. These findings are similar to those of studies in people living with HAE, where a rating of “a little better” for two consecutive time points can support the “onset of symptom relief” definition (32). In light of the findings from this study, a PGI-C rating of at least “better” sustained at 12 hours post treatment was selected as the primary endpoint of a phase 3 trial investigating deucrictibant for on-demand treatment of AAE-C1INH attacks. Secondary endpoints in this trial include “time to complete resolution” defined as a PGI-S rating of “no symptoms” sustained within 24 hours post treatment, which was deemed the only meaningful outcome at 24 hours post treatment by participants of this study. Embedded interviews will also be conducted during this upcoming trial and will focus on participants’ perceptions of meaningful within-patient changes on the PGI-C and PGI-S.

Overall, our study confirmed that AAE-C1INH can have serious impacts on people’s lives and demonstrated that PGI and PGA scales can be used in this population to evaluate perception of treatment outcomes. This study has several strengths. Of note, it is the first in-depth, qualitative study to assess the experiences of individuals with AAE-C1INH and to validate potentially relevant PRO items. We conducted direct participant interviews to ensure that outcomes were grounded in their own experiences. This aligns with US FDA guidance on patient-focused drug development, which provides a framework for collecting and using patient-centered data in the drug development process (33). Although AAE-C1INH is a rare disease, we collected an array of multifaceted data about symptoms, disease impacts, and feedback for PROs that can be built upon in future studies. These findings had immediate translational impact as they directly informed the design of the first phase 3 trial in AAE-C1INH—the randomized, placebo-controlled, phase 3 CREAATE trial—exemplifying how patient input can help shape meaningful trial endpoints.

Limitations of this study include the small number of participants and that most participants were female, all were living in the US, and all had a high level of education, which may affect the generalizability of the findings to a broader population of individuals with AAE-C1INH. Participants were recruited through a patient advocacy organization, which may have introduced selection bias toward more highly motivated or knowledgeable individuals. However, participants had a median age of symptom onset of approximately 56 years, which is comparable to other reports (4, 5). Despite the small sample size, owing to the rarity of the disease, and the challenges identifying and enrolling eligible participants, individuals provided rich descriptions of their disease journey and treatment experiences that informed development of a conceptual model of AAE-C1INH. Concept saturation was not reached in this study, aligning with FDA Patient-Focused Drug Development guidance, which recognizes that achieving saturation can be particularly challenging in the context of rare diseases. Despite not reaching saturation, participants described 57 unique subconcepts across 10 different attack areas, thereby contributing to our understanding of AAE-C1INH. To examine whether further symptoms or physical manifestations may be present in this population and to confirm the findings in this study, larger studies with more heterogeneous populations are warranted. Additionally, underlying disorders associated with AAE-C1INH can independently impact patients’ lives leading to further challenges for patients, including healthcare providers’ unfamiliarity with AAE-C1INH, delays in diagnosis and treatment, uncertainty about future wellbeing, and the potential burden of treatment expenses. Therefore, it is often difficult to distinguish the impairments caused by the underlying condition from those caused by the AAE-C1INH swellings themselves.

Overall, this qualitative study underscores the rarity of AAE−C1INH and the profound unmet needs experienced by people living with the disease. Findings from this study suggest that the unpredictability and severe nature of angioedema attacks profoundly impacted daily functioning and were exacerbated by misdiagnoses and the lack of treatment options for individuals with AAE-C1INH. By developing a conceptual model of AAE-C1INH and providing the first evidence on the relevance and potential suitability of PGI and PGA items in individuals with AAE-C1INH, this study has informed the design of the first phase 3 clinical trial of a potential prophylactic and on-demand treatment for AAE-C1INH attacks.

## Data Availability

Pharvaris will consider requests from qualified scientific and medical researchers to share deidentified participant data that underlie results in this Article. Data requests will be considered from 6 months after marketing approval of study drug in both the USA and European Union. Supporting documents, including the study protocol and statistical analysis plan, can also be requested. Proposed research should seek to answer a previously unanswered important medical or scientific questions and any data-sharing requests should reflect those important questions. Any applicable country-specific privacy and other laws and regulations will be considered and might prevent sharing of data. For further information on the process and requirements for submitting a data-sharing request, please contact Pharvaris at DSR@pharvaris.com.

## References

[1] Grumach AS Veronez CL Csuka D Farkas H . Angioedema without wheals: challenges in laboratorial diagnosis. Front Immunol. (2021) 12:785736. doi: 10.3389/fimmu.2021.785736. PMID: 34956216 PMC8694242

[2] Swanson TJ Patel BC . Acquired angioedema. In: StatPearls [Internet]. Treasure Island (FL): StatPearls Publishing (2026). Available online at: https://www.ncbi.nlm.nih.gov/books/NBK430889/. 28613639

[3] Shi Y Wang C . Where we are with acquired angioedema due to C1 inhibitor deficiency: a systematic literature review. Clin Immunol. (2021) 230:108819. doi: 10.1016/j.clim.2021.108819. PMID: 34358691

[4] Pólai Z Balla Z Andrási N Kőhalmi K Temesszentandrási G Benedek S . A follow‐up survey of patients with acquired angioedema due to C1‐inhibitor deficiency. J Intern Med. (2021) 289:547–58. doi: 10.1111/joim.13182. PMID: 33215769

[5] Trainotti S Johnson F Hahn J Hofauer B Greve J Wollenberg B . Acquired angioedema due to C1-inhibitor deficiency (AAE-C1-INH)-a bicenter retrospective study on diagnosis, course, and therapy. J Allergy Clin Immunol Pract. (2023) 11:3772–9. doi: 10.1016/j.jaip.2023.09.003. PMID: 37716525

[6] Gobert D Paule R Ponard D Levy P Frémeaux-Bacchi V Bouillet L . A nationwide study of acquired C1-inhibitor deficiency in France: characteristics and treatment responses in 92 patients. Med (Baltimore). (2016) 95:e4363. doi: 10.1097/MD.0000000000004363. PMID: 27537564 PMC5370791

[7] Fernandez J . Hereditary and acquired C1 inhibitor deficiency (2024). Available online at: https://www.merckmanuals.com/home/immune-disorders/allergic-reactions-and-other-hypersensitivity-disorders/hereditary-and-acquired-c1-inhibitor-deficiency (Accessed January 13, 2026).

[8] Wagner WO . Angioedema: frightening and frustrating. Cleve Clin J Med. (1999) 66:203–5. doi: 10.3949/ccjm.66.4.203. PMID: 10199054

[9] Petersen RS Fijen LM Kelder JP Cohn DM . Deucrictibant for angioedema due to acquired C1-inhibitor deficiency: a randomized-controlled trial. J Allergy Clin Immunol. (2024) 154:179–83. doi: 10.1016/j.jaci.2024.03.007. PMID: 38494092

[10] Longhurst H Zanichelli A Caballero T Bouillet L Aberer W Maurer M . Comparing acquired angioedema with hereditary angioedema (types I/II): findings from the Icatibant Outcome Survey. Clin Exp Immunol. (2017) 188:148–53. doi: 10.1111/cei.12910. PMID: 27936514 PMC5343339

[11] Cicardi M Zanichelli A . Acquired angioedema. Allergy Asthma Clin Immunol. (2010) 6:14. doi: 10.1186/1710-1492-6-14. PMID: 20667117 PMC2925362

[12] Sobotkova M Zachova R Hakl R Kuklinek P Kralickova P Krcmova I . Acquired angioedema with C1 inhibitor deficiency: occurrence, clinical features, and management: a nationwide retrospective study in the Czech Republic patients. Int Arch Allergy Immunol. (2021) 182:642–9. doi: 10.1159/000512933. PMID: 33472202 PMC8315685

[13] Baeza ML González-Quevedo T Caballero T Guilarte M Lleonart R Varela S . Angioedema due to acquired deficiency of C1-inhibitor: a cohort study in Spain and a comparison with other series. J Allergy Clin Immunol Pract. (2022) 10:1020–8. doi: 10.1016/j.jaip.2021.11.018. PMID: 34844023

[14] Belbézier A Boccon-Gibod I Bouillet L . Efficacy of lanadelumab in acquired angioedema with C1-inhibitor deficiency. J Allergy Clin Immunol Pract. (2021) 9:2490–1. doi: 10.1016/j.jaip.2021.01.040. PMID: 33556593

[15] Johnson F Stenzl A Hofauer B Heppt H Ebert EV Wollenberg B . A retrospective analysis of long-term prophylaxis with berotralstat in patients with hereditary angioedema and acquired C1-inhibitor deficiency-real-world data. Clin Rev Allergy Immunol. (2023) 65:354–64. doi: 10.1007/s12016-023-08972-2. PMID: 37914894 PMC10847220

[16] Zanichelli A Bova M Coerezza A Petraroli A Triggiani M Cicardi M . Icatibant treatment for acquired C1-inhibitor deficiency: a real-world observational study. Allergy. (2012) 67:1074–7. doi: 10.1111/j.1398-9995.2012.02853.x. PMID: 22686628

[17] Aygören-Pürsün E Stobiecki M Valerieva A Cancian M Gompels M Grigoriadou S . CHAPTER-1 trial. Lancet Haematol. (2026) 13:e215-26. doi: 10.1016/S2352-3026(26)00004-9 41865746

[18] Maurer M Stobiecki M Valerieva A Hakl R Staevska MT Bouillet L . CHAPTER-1 trial. Lancet Haematol. (2026) 13:e200-14. doi: 10.1016/S2352-3026(25)00341-2 41865747

[19] de Lange M Petersen RS FIjen LM Cohn DM . Long-term prophylactic treatment with deucrictibant for angioedema due to acquired C1-inhibitor deficiency. J Allergy Clin Immunol. (2025) 156:1650–5. doi: 10.1016/j.jaci.2025.07.033. PMID: 40882771

[20] US Food and Drug Administration . Orphan drug designations and approvals: treatment of bradykinin-mediated angioedema (2022). Available online at: https://www.accessdata.fda.gov/scripts/opdlisting/oopd/detailedIndex.cfm?cfgridkey=809521 (Accessed December 19, 2025).

[21] Brix ATH Boysen HB Weller K Caballero T Bygum A . Patient-reported outcome measures for angioedema: a literature review. Acta Derm Venereol. (2021) 101:adv00456. doi: 10.2340/00015555-3807. PMID: 33880569 PMC9367035

[22] Vanya M Watt M Shahraz S Kosmas CE Rhoten S Costa-Cabral S . Content validation and psychometric evaluation of the Angioedema Quality of Life Questionnaire for hereditary angioedema. J Patient Rep Outcomes. (2023) 7:33. doi: 10.1186/s41687-023-00576-w. PMID: 37012445 PMC10070575

[23] Weller K Groffik A Magerl M Tohme N Martus P Krause K . Development and construct validation of the angioedema quality of life questionnaire. Allergy. (2012) 67:1289–98. doi: 10.1111/all.12007. PMID: 22913638

[24] Weller K Groffik A Magerl M Tohme N Martus P Krause K . Development, validation, and initial results of the Angioedema Activity Score. Allergy. (2013) 68:1185–92. doi: 10.1111/all.12209. PMID: 23919330

[25] Braun V Clarke V . Using thematic analysis in psychology. Qual Res Psychol. (2006) 3:77–101. doi: 10.1191/1478088706qp063oa. PMID: 32100154

[26] Joffe H Yardley L . Content and thematic analysis. In: Marks D Yardley L , editors.Research Methods for Clinical and Health Psychology. Sage Publications, Ltd, London, UK (2004). p. 56–68.

[27] Bryant A Charmaz K . The SAGE Handbook of Current Developments in Grounded Theory. London, UK: Sage Publications Ltd (2019). p. 714.

[28] Corbin J Strauss A . Basics of Qualitative Research: Techniques and Procedures for Developing Grounded Theory. Thousand Oaks, CA: Sage Publications, Inc (2015).

[29] Patrick DL Burke LB Gwaltney CJ Leidy NK Martin ML Molsen E . Content validity--establishing and reporting the evidence in newly developed patient-reported outcomes (PRO) instruments for medical product evaluation: ISPOR PRO good research practices task force report: part 1-- eliciting concepts for a new PRO instrument. Value Health. (2011) 14:967–77. doi: 10.1016/j.jval.2011.06.014. PMID: 22152165

[30] Kerr C Nixon A Wild D . Assessing and demonstrating data saturation in qualitative inquiry supporting patient-reported outcomes research. Expert Rev Pharmacoecon Outcomes Res. (2010) 10:269–81. doi: 10.1586/erp.10.30. PMID: 20545592

[31] Zanichelli A Azin GM Wu MA Suffritti C Maggioni L Caccia S . Diagnosis, course, and management of angioedema in patients with acquired C1-inhibitor deficiency. J Allergy Clin Immunol Pract. (2017) 5:1307–13. doi: 10.1016/j.jaip.2016.12.032. PMID: 28284781

[32] Riedl MA Farkas H Aygören-Pürsün E Psarros F Soteres DF Staevska M . Oral sebetralstat for on-demand treatment of hereditary angioedema attacks. N Engl J Med. (2024) 391:32–43. doi: 10.1056/NEJMoa2314192. PMID: 38819658

[33] US Food and Drug Administration . Patient-focused drug development: incorporating clinical outcome assessments into endpoints for regulatory decision-making; draft guidance for industry, Food and Drug Administration staff, and other stakeholders; availability (2023). Available online at: https://www.federalregister.gov/documents/2023/04/06/2023-07243/patient-focused-drug-development-incorporating-clinical-outcome-assessments-into-endpoints-for# (Accessed December 19, 2025).

