# Supplementary material for: Angioedema due to acquired C1 inhibitor deficiency: patient experience, conceptual disease model, and assessment of patient-reported outcome measures
Source: Front Immunol. 2026 May 14;17:1810851. doi: 10.3389/fimmu.2026.1810851 (PMC13215923; doi:10.3389/fimmu.2026.1810851)
Supplement: Supplementary file 1 [file DataSheet1.docx]

# Supplementary Material

**Supplementary text**

## Full list of eligibility criteria

Inclusion criteria were:

- Adults aged ≥18 years at the time of providing written informed consent/assent
- Diagnosis of AAE-C1INH based upon all of the following:
  - Documented clinical history consistent with AAE-C1INH (subcutaneous or mucosal, nonpruritic swelling without accompanying urticaria)
  - Diagnostic testing results to confirm AAE-C1INH
    - C1-INH functional level <40% of the normal level
  - No family history of an angioedema diagnosis
  - And at least one of the following:
    - Age ≥40 years at reported onset of first angioedema symptoms
    - C1q is below the lower limit of the normal range
    - Serological confirmation of anti-C1-inhibitor antibodies
- At least one AAE attack in the last 3 months (12 weeks)
  - *Note: If recruitment is deemed to be too difficult due to this criterion, the study team may relax this criterion to allow participants who have had at least one attack in the last 6 months*
- Stable underlying disease of AAE-C1INH (e.g., lymphoproliferative disease, immune complex disorders, monoclonal gammopathy of undetermined significance) – specifically, treatment for the underlying disease causing AAE-C1INH has not changed for the last 3 months and can be reasonably expected to remain unchanged for the next 6 months
- Willing and able to take part in a 90-minute interview in English
- Willing to have the interview audio-recorded
- Has access to the internet and a computer or tablet to participate in the study

Due to difficulties with recruitment because of the rarity of the condition, the Sponsor extended the criteria to allow for participants with slightly higher C1-INH functional levels and less stable underlying disease, both to be determined at the discretion of the study team. These revisions were approved by the IRB. Specific wording of extended criteria:

- Extended criterion: Participants with C1-INH functional level >40% may be allowed at the discretion of the study team
- Extended criterion: Participants with unstable underlying disease may be permitted into the study at the discretion of the study team

Exclusion criteria were:

- Any prior or concomitant diagnosis of angioedema other than AAE-C1INH
- Does not wish to or unable to take part in a 90-minute interview
- Does not read, write, or speak English fluently
- Does not have access to the internet
- Does not have access to a computer or tablet
- Does not wish to have the interview audio-recorded

## Descriptions of attacks based on location

*Abdominal*

Participants described the characteristics of attacks based on attack location. Nearly all interviewed participants (n=7, 87.5%) reported experiencing swelling attacks in their intestines or abdominal area.

“So, it’s not so much a visual, like I have a swelling, if I had a swelling in my extremities, but you have a very painful experience in your abdominal area, almost like appendicitis or something. It’s just very painful because everything is being pushed together.” [Participant 8]

Approximately one-third of participants (n=3, 37.5%) reported experiencing nausea, intestinal dysfunction (the inability to pass stool), and/or severe fatigue during – or as a precursor to – an intestinal attack.

“Almost nausea all the time, and then, when you get an attack, you can have a mild attack where you’re really tired, a little nausea, and I don’t get swelling in the way they talk about it.” [Participant 4]

*Facial edema*

Six participants (75.0%) reported facial edema. It was typically described as swelling of the lips (Participants 1, 6, 7, and 8), cheeks (Participants 1 and 4), and eyes (Participants 5 and 8).

“I usually swell – around my lips would swell and my cheek would swell, and I had some pain with it, disco – extreme discomfort more than pain, and it’s just embarrassment ‘cause it just looks awful.” [Participant 8]

“Okay, so, about 12 years ago, I had my first episode of swelling in my face, and we went to the local Emergency Room and [laughs] when you walk into the Emergency Room looking like I did, people start scuttling around quite a bit.” [Participant 1]

*Foot edema*

Five participants (62.5%) discussed experiencing foot edema. The triggers for swelling varied, often attributed to specific activities. For instance, participant two noted that their feet started swelling after dancing at a wedding.

“Well, it could be pretty severe. I mean, I often had to have someone come and pick me up from work. I couldn’t drive and you can’t really walk around your house. I had a dog at the time and had to have somebody come over to take my dog out ‘cause I couldn’t do it.” [Participant 8]

*Hand edema*

Five participants (62.5%) reported experiencing hand edema. Three participants (Participants 2, 4, and 7) described the changed appearance of their hand due to the swelling.

“It’s like in between my wrist and my elbow, like a center area, all of a sudden, it gets hard. It gets hard and it doesn’t feel right. Like you would like it’d be a bug bite, do you know? Like, it doesn’t get red or discolored. It just – it – the texture feels different, you know, and then, it, kind of, grows, it, kind of, expands. It – like, you know, and that’s where it might go into my hand or, like – and my – or, like, if it’s my right hand, my right hand’s my writing hand, you know, and, you know, that can make things very difficult at work and, you know, for me to function, yeah.” *[Participant 2]*

*Throat edema*

Throat edema was reported by half of participants (n=4, 50.0%). The throat swelling was described in various ways by participants. Descriptions included being “lower in my throat” (Participant 1), feeling “heavy” (Participant 3), and “laryngeal swelling” (Participants 4 and 7).

“It’s the ones where I see, like, it swelling internally. If I open my mouth and I see that closing up at the back of my mouth, that’s the ones where I, like, really, really worry.” [Participant 3]

*Tongue edema*

Tongue edema was reported by three participants (37.5%) and was described as swelling in the tongue by all participants.

“‘Cause it was, like – it was horrible, ‘cause you couldn’t talk, I couldn’t talk ‘cause my tongue was so big, you know. So, I couldn’t talk to them and then, they’re, like, treating me like a psych case. It was just – it was so traumatic.” [Participant 3]

*Genital edema*

Genital edema was reported by three of eight participants (37.5%). This was described as swelling in the genital area, with two participants referencing the “*reproductive area*” (Participant 1) or “*private parts*” (Participant 8).

“…I had some urinary retention with that, so unable to urinate, and that went away by itself over the day.” [Participant 4]

*Arm edema*

Arm edema was also reported by three participants (37.5%). Two participants (Participant 2 and 7) also mentioned swelling in their arms, moving either down to their hands or from their hands to their arms.

“Then, I carried on with my day and I noticed it [arm] was getting a little larger, a little bigger, harder, and it was going into my hand.” [Participant 2]

*Neck edema*

Neck edema was reported by three participants (37.5%) and described as neck swelling by all participants. Neck swelling was differentiated from throat swelling due to it being “outward as opposed to inward” (Participant 3).

“That’s actually – like, I feel like that’s actually skin fluid as opposed to internal, so it doesn’t bother me as much, you know, because – I mean, it looks really weird…” [Participant 3]

*Joint edema*

Only one participant (12.5%) (Participant 5) reported experiencing joint swelling. They described their joints as “stiff and sore” for days or even weeks, feeling as though they had “aged decades.”

“So, it’s a very steep stairwell going up there, and I go up each day because I ride the exercise bike every day, and it was all I could do to make it up those stairs. It was that stiff and sore, and I just felt like I’d aged decades, you know…” [Participant 5]

## Supplementary figures

**
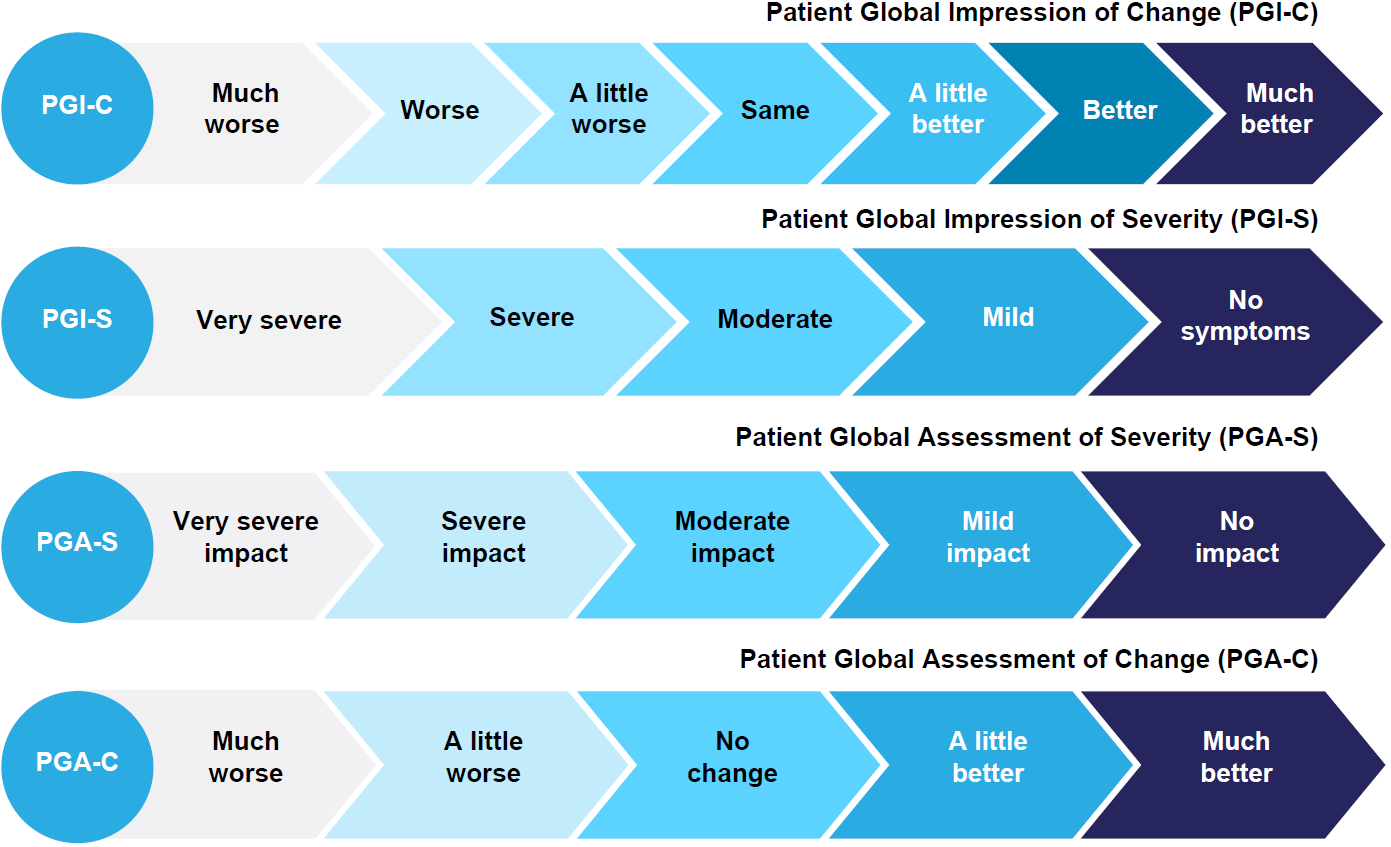
**

**Supplementary Figure 1.** Clinical outcome assessment scales for Patient Global Impression and Patient Global Assessments.

**SUPPLEMENTARY Table 1** Saturation grid.

| **Attack areas and related symptoms/physical impacts** | **n (%)** | **1**  **26- Nov-24** | **2**  **01-Dec-24** | **3**  **06-Dec-24** | **4**  **06-Dec-24** | **5**  **06-Dec-24** | **6**  **10-Dec-24** | **7**  **13-Dec-24** | **8**  **18-Dec-24** |
| --- | --- | --- | --- | --- | --- | --- | --- | --- | --- |
| **Intestinal/abdominal edema** | 7 (87.5) |  | √ | √ | √ | √ | √ | √ | √ |
| Abdominal pain | 5 (62.5) |  | √ |  |  | √ | √ | √ | √ |
| Bloating | 5 (62.5) |  |  | √ | √ | √ | √ | √ |  |
| Impacts on eating and drinking | 5 (62.5) |  | √ |  | √ |  | √ | √ | √ |
| Vomiting | 4 (50.0) |  | √ |  | √ |  | √ | √ |  |
| Intestinal dysfunction/  inability to go | 3 (37.5) |  | √ |  | √ | √ |  |  |  |
| Nausea | 3 (37.5) |  |  |  | √ |  |  | √ | √ |
| Fatigue/weak/“wiped out” | 3 (37.5) |  |  |  | √ |  | √ | √ |  |
| Diarrhea/loose bowel movements | 2 (25.0) |  | √ |  |  | √ |  |  |  |
| Abdominal discomfort | 2 (25.0) |  | √ |  | √ |  |  |  |  |
| Bedridden | 2 (25.0) |  |  |  |  |  | √ |  | √ |
| Difficulty sleeping/lost sleep | 2 (25.0) |  |  |  |  |  | √ | √ |  |
| Mobility impacts | 2 (25.0) |  | √ |  |  |  |  | √ |  |
| Feeling full | 1 (12.5) |  |  |  | √ |  |  |  |  |
| Dry heaving | 1 (12.5) |  |  |  |  |  |  | √ |  |
| Cold sweat | 1 (12.5) |  |  |  |  |  |  | √ |  |
| Listless | 1 (12.5) |  |  |  |  | √ |  |  |  |
| Gas | 1 (12.5) |  |  |  |  | √ |  |  |  |
| “Intestinal pressure” | 1 (12.5) |  |  |  |  | √ |  |  |  |
| Cramping | 1 (12.5) |  |  |  |  | √ |  |  |  |
| Burping | 1 (12.5) |  |  |  | √ |  |  |  |  |
| Dehydrated | 1 (12.5) |  |  |  |  |  |  | √ |  |
| **Facial edema** | 6 (75.0) | √ |  | √ | √ |  | √ | √ | √ |
| Lip edema | 4 (50.0) | √ |  |  |  |  | √ | √ | √ |
| Eye edema | 2 (25.0) |  |  |  |  | √ |  |  | √ |
| Alters appearance | 3 (37.5) | √ |  |  | √ |  |  | √ |  |
| Need to rest | 1 (12.5) | √ |  |  |  |  |  |  |  |
| Whole head swollen | 1 (12.5) |  |  |  |  |  | √ |  |  |
| Eye bruising | 1 (12.5) |  |  |  |  | √ |  |  |  |
| Tingling | 1 (12.5) | √ |  |  |  |  |  |  |  |
| Skin tightness/pressure | 1 (12.5) | √ |  |  |  |  |  |  |  |
| Unable to breathe through nose | 1 (12.5) |  |  | √ |  |  |  |  |  |
| **Foot edema** | 5 (62.5) | √ | √ |  | √ |  |  | √ | √ |
| Difficulty walking | 2 (25.0) |  | √ |  |  |  |  |  | √ |
| Difficulty wearing shoes | 2 (25.0) |  |  |  |  |  |  | √ | √ |
| Pain | 1 (12.5) |  |  |  |  |  |  |  | √ |
| **Hand edema** | 5 (62.5) | √ | √ |  | √ |  |  | √ | √ |
| Difficulty using hands | 2 (25.0) |  | √ |  |  |  |  | √ |  |
| Dryness/cracking | 1 (12.5) |  |  |  | √ |  |  |  |  |
| **Throat edema** | 4 (50.0) | √ |  | √ | √ |  |  | √ |  |
| Voice changes | 2 (25.0) | √ |  |  | √ |  |  |  |  |
| Unable to breathe | 1 (12.5) |  |  |  | √ |  |  |  |  |
| Feeling of something stuck   in throat | 1 (12.5) |  |  |  |  |  |  | √ |  |
| Can't swallow water   (“shot out of mouth”) | 1 (12.5) |  |  | √ |  |  |  |  |  |
| Heavy feeling | 1 (12.5) |  |  | √ |  |  |  |  |  |
| “Closing up at back of mouth” | 1 (12.5) |  |  | √ |  |  |  |  |  |
| Pressing/swelling inward | 1 (12.5) |  |  | √ |  |  |  |  |  |
| **Tongue edema** | 3 (37.5) | √ |  | √ |  | √ |  |  |  |
| Can't talk | 1 (12.5) |  |  | √ |  |  |  |  |  |
| Numbness | 1 (12.5) |  |  |  |  | √ |  |  |  |
| Drooling | 1 (12.5) |  |  | √ |  |  |  |  |  |
| **Genital edema** | 3 (37.5) | √ |  |  | √ |  |  |  | √ |
| Buttocks edema | 1 (12.5) |  |  |  |  |  |  |  | √ |
| Scrotum edema | 1 (12.5) | √ |  |  |  |  |  |  |  |
| Difficulty urinating | 1 (12.5) |  |  |  | √ |  |  |  |  |
| Tenderness | 1 (12.5) | √ |  |  |  |  |  |  |  |
| **Arm edema** | 3 (37.5) |  | √ |  |  |  |  | √ | √ |
| Hardness/thickness | 1 (12.5) |  | √ |  |  |  |  |  |  |
| Flu-like symptoms before swelling (fever, sweating, weakness) | 1 (12.5) |  |  |  |  |  |  |  | √ |
| **Neck edema** | 3 (37.5) | √ |  | √ |  |  |  |  | √ |
| “Looks really weird” | 1 (12.5) |  |  | √ |  |  |  |  |  |
| **Joint edema** | 1 (12.5) |  |  |  |  | √ |  |  |  |
| Joint stiffness/soreness | 1 (12.5) |  |  |  |  | √ |  |  |  |
| Walked like a zombie | 1 (12.5) |  |  |  |  | √ |  |  |  |
| Difficulty walking up stairs | 1 (12.5) |  |  |  |  | √ |  |  |  |
| **Body rash (not related to   specific attack area)** | 1 (12.5) |  |  |  |  | √ |  |  |  |
| **Fatigue (not related to   specific attack area)** | 1 (12.5) |  |  |  | √ |  |  |  |  |
| **Total number of core attack   areas per participant** |  | 7 | 4 | 5 | 6 | 3 | 2 | 6 | 7 |
| **Total number of   subconcepts/symptoms** |  | 8 | 10 | 9 | 15 | 15 | 9 | 16 | 11 |
| **Total number of core attack   areas across participants (N=10)** | **New areas per participant** | 7 | 2 | 0 | 0 | 1 | 0 | 0 | 0 |
| **Total number of subconcepts   across participants (N=57)** | **New subconcepts per participant** | 8 | 10 | 9 | 8 | 11 | 3 | 5 | 3 |
| **Cumulative number of attack   areas applied** |  | 7 | 9 | 9 | 9 | 10 | 10 | 10 | 10 |
| **Cumulative number of   subconcepts applied** |  | 8 | 18 | 27 | 35 | 46 | 49 | 54 | 57 |
| **Cumulative percentage of   attack areas applied** |  | 70.0 | 90.0 | 90.0 | 90.0 | 100 | 100 | 100 | 100 |
| **Cumulative percentage of   subconcepts applied** |  | 14.0 | 31.6 | 47.4 | 61.4 | 80.7 | 86.0 | 94.7 | 100 |

“√” indicates attack areas and related symptoms/physical impacts experienced.
